# Repeatability of the human eye compared to an intraoral scanner in dental shade matching

**DOI:** 10.1016/j.heliyon.2019.e02100

**Published:** 2019-07-23

**Authors:** Juan Reyes, Pamela Acosta, Dalina Ventura

**Affiliations:** Alumnus, Stomatology School, Pontificia Universidad Católica Madre y Maestra, Santiago de los Caballeros, Santiago, Dominican Republic

**Keywords:** Dentistry, Repeatability, Color dimensions, Tooth colour, Intraoral scanner, Dental shade matching

## Abstract

**Objective:**

The purpose of this investigation was to compare the repeatability of an intraoral scanner (3Shape TRIOS) with the traditional visual method for dental shade matching in patients and to assess the influence of ambient lighting and the observer's sex and experience on visual shade matching. An additional aim was to determine the color dimension for which repeatability is greater with both the visual method and intraoral scanner.

**Methods:**

Thirty observers (15 men and 15 women), grouped by professional experience, selected the shade of the right maxillary central incisor in 10 patients on three different occasions under different ambient lighting conditions (twice under studio clinic lighting and once under natural light). The same procedure was repeated using an intraoral scanner. All shades were selected based on the VITA Toothguide 3D-MASTER. The repeatability of each observer and the intraoral scanner were recorded for each color dimension (hue, chroma, and value).

**Results:**

The TRIOS intraoral scanner obtained a mean repeatability of 86.66% in dental shade matching compared to 75.22% achieved by the visual method. Ambient lighting had a direct effect on the repeatability of the shade selection for the visual method, whereas the observer's sex and clinical experience did not. For the visual method, the repeatability in dental shade matching depended on the dimension studied, with the best results in value, followed by hue and chroma; however, such dependence was not detected for the intraoral scanner.

**Conclusions:**

The TRIOS intraoral scanner ensured better repeatability than the visual method in dental shade matching.

## 1 Introduction

Aesthetics has gradually become a very important factor in the lives of people. Dentistry is not indifferent to this reality; in fact, aesthetic considerations are a determining factor in the choice of a practitioner. Today, one of the biggest challenges of the profession is to address the needs of patients by achieving the highest possible aesthetic level.

Dental prostheses, defined as elements manufactured with the purpose of replacing one or more dental pieces lost for different reasons, represent the area of dentistry where aesthetics plays the most important role, especially in the maxillary anterior sector. A fundamental aesthetic factor is determining the right color for the prosthesis [1].

Perception of color is a complex physiological mechanism, in which our brain interprets electric signals coming from specialized cells of the eye, previously stimulated by the light rays reflected by the environment. However, this mechanism is not infallible, and the way people appreciate color and its components can be influenced by various factors, including the person's own subjectivity, fatigue, mood, and environmental factors, such as lighting and the phenomenon of metamerism [2].

To assess the color of a dental piece, dentistry offers two general methods: visual and instrumental. The first is based on a comparison of the patient's own color with the dental shade guides available in the market; the operator selects the tone that seems most appropriate, according to his or her clinical criteria. These shade guides have often been criticized and suggestions have been made for their improvement [3, 4, 5]. Following these suggestions, VITA introduced a new shade matching guide with a greater number of tabs than those included in the older Classic model; it is arranged in a more logical way and takes into account the three dimensions of color: hue, chroma, and value [1]. With the implementation of these changes, the newly introduced VITA Toothguide 3D-MASTER showed better results than its predecessor [6, 7].

The second method is based on instruments developed with the purpose of overcoming the limits of the visual method, particularly the dependence on personal or environmental factors. Within this group is the spectrophotometer, which is considered the gold standard in dental color screening research. In addition, colorimeters, spectroradiometers, software photo analysis, and intraoral scanners are used for this purpose. The findings of some previous studies agree on the better performance of instrumental means in both accuracy and reproducibility when compared to visual shade matching [1, 8, 9, 10, 11, 12, 13, 14, 15, 16, 17, 18, 19]. However, both the visual and instrumental methods use shade guides as references, which often fail to faithfully represent the polychromatic nature of tooth color, and the results obtained vary according to the brand and the material used to manufacture the prosthesis [7, 20].

Regarding the observer's experience, Della Bona et al. [6] found a greater correlation between the visual and the instrumental method when the observers were experienced. Their ideas are supported by other studies conducted in different conditions and scenarios [21, 22, 23]. However, many authors like Kröger et al. [8] claim that the differences between groups of observers based on clinical experience were minimal and of no practical importance. Other studies reached the same conclusion, arguing that when using the VITA Toothguide 3D-MASTER, experience was not an influential factor in the selection of dental color [24, 25, 26].

Lighting, an essential factor in our ability to differentiate colors, has been described as inducing differences in the selection of a dental tone between observers [16, 21, 27]. Despite this fact, Wee et al. [28] warned the dental community that most of the offices did not meet the basic lighting requirements for the correct selection of tooth color.

The sex of the observer has often been postulated to be an element potentially influencing repeatability in choosing the tone of a tooth. Sometimes, as demonstrated by Haddad et al. [29], women have achieved better results than men. In other cases, such as in the investigation by Miranda et al. [30], the opposite result was found. Several studies have cast doubt on the reality of such differences due to the inconsistency of the results [22, 24, 25, 31, 32].

Three dimensions have been proposed to identify colors — hue, chroma, and value — with different levels of clinical relevance in dentistry. The shade guides widely used in dental practice do not allow each dimension to be distinguished individually. However, this limitation has been overcome with the development of the VITA Toothguide 3D-MASTER, and investigators have been able to evaluate the incidence of each dimension of color in shade matching, although with experimental designs, statistical methods, and outcomes different from those adopted in this study. Some studies, such as the one by Gómez-Polo et al. [33], observed a greater correlation between visual and instrumental methods in terms of the value, followed by hue and finally chroma. This ordering agrees with the respective clinical importance of the three dimensions in obtaining an adequate result in shade selection for dental prostheses.

Intraoral scanners were initially designed to produce instantaneous digital dental impressions. However, newer models, such as the 3Shape TRIOS, are capable of simultaneously selecting dental color by means of a high-definition camera, LED light, computer software, and the use of the VITA shade guide as a reference [11]. In 2018, Moussaoui [34] published an analysis of the literature, in which he identified only three in vivo studies comparing the 3Shape TRIOS intraoral scanner with the visual method or other instrumental methods in dental shade matching. The findings of these studies suggested that the TRIOS intraoral scanner could be used as an alternative to the visual method [11, 35, 36]. In some cases, the results were comparable to those obtained using the spectrophotometer, the instrument considered to be the current gold standard [11, 35].

Most of the studies mentioned above were conducted in vitro and considered the results obtained by the instrumental methods, usually a spectrophotometer, as the gold standard for statistical purposes. In certain cases, only a measurement coinciding exactly with that obtained by the device was considered correct, thus discarding those that coincided in some but not all the color dimensions.

To overcome such limitations, the aim of this study was to evaluate the repeatability of dental shade selection by comparing the visual method and the 3Shape TRIOS intraoral scanner, and to evaluate the effect of ambient lighting and the observer's sex and experience level in using the visual method. Additionally, the repeatability reached by each method was separately assessed with regard to the three dimensions of color. It is important to note that the repeatability of an instrument is related to its ability to produce the same results over time in measurements made under the same conditions as the original measurement. In contrast, the accuracy of an instrument is calculated by comparing its measurements with an instrument considered to be a gold standard [34]. Both qualities are sought in an ideal color measuring instrument. The purpose of this research was to assess the repeatability of the two methods of color selection. The main hypothesis was that the intraoral scanner would achieve higher repeatability than the visual method in dental shade matching. Other hypotheses were that the visual method would be influenced by ambient lighting, and by the sex and the experience of the observer.

## 2 Materials and methods

A longitudinal, analytical, and quasi-experimental study was performed. It was submitted for consideration and was examined and retrospectively approved by the Bioethics Committee of the Faculty of Health Sciences of the Pontificia Universidad Católica Madre y Maestra - CSTI. Forty participants were selected (30 examiners and 10 patients). Written informed consent was obtained from each participant for participation in the study and further use of the data and pictures collected during the study for academic purposes. The examiners were classified into three groups according to their expertise level. The first group (10 examiners) comprised 4th year students from the School of Dental Medicine, the second group (10) comprised 5th year students, and the third group comprised 10 prosthodontists. The examiners were screened by their primary practitioners for any color vision deficiencies using the Ishihara color vision test. Participants with a visual impairment of any kind were excluded from the study. The examiners were instructed on the proper handling of the VITA Toothguide 3D-MASTER as per the shade guide's instruction manual. The patients, who served as models, had upper central incisors in optimal conditions, without any type of restoration, prosthesis, or previous whitening treatment. The study evaluated the examiners' repeatability in selecting dental shades using the visual method, with regard to their experience, sex, and the ambient lighting used. As for the instrumental method, only the influence of ambient lighting was evaluated.

**Fig. 1:**
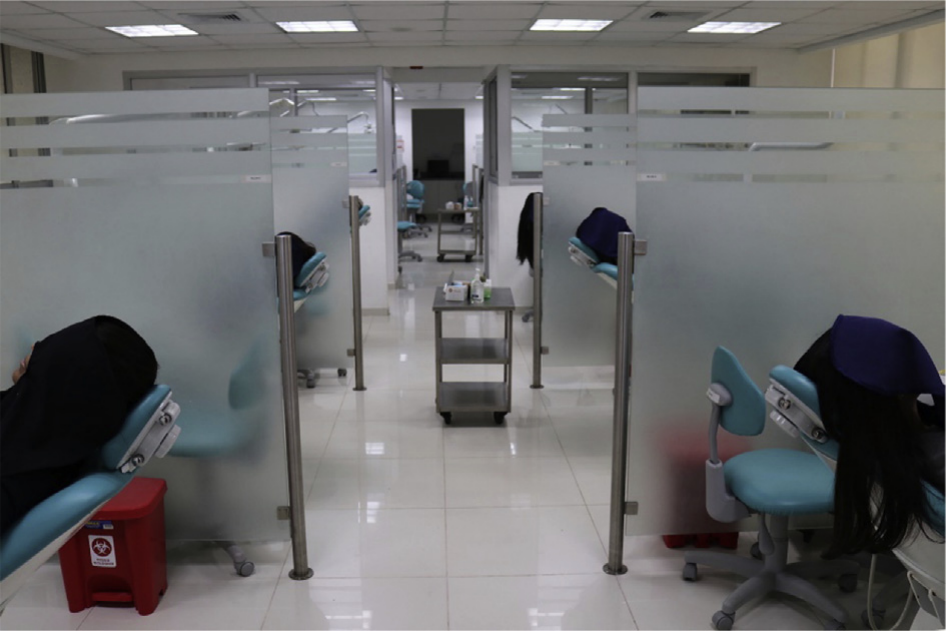
Disposition of the patients in the clinic.

**Fig. 2:**
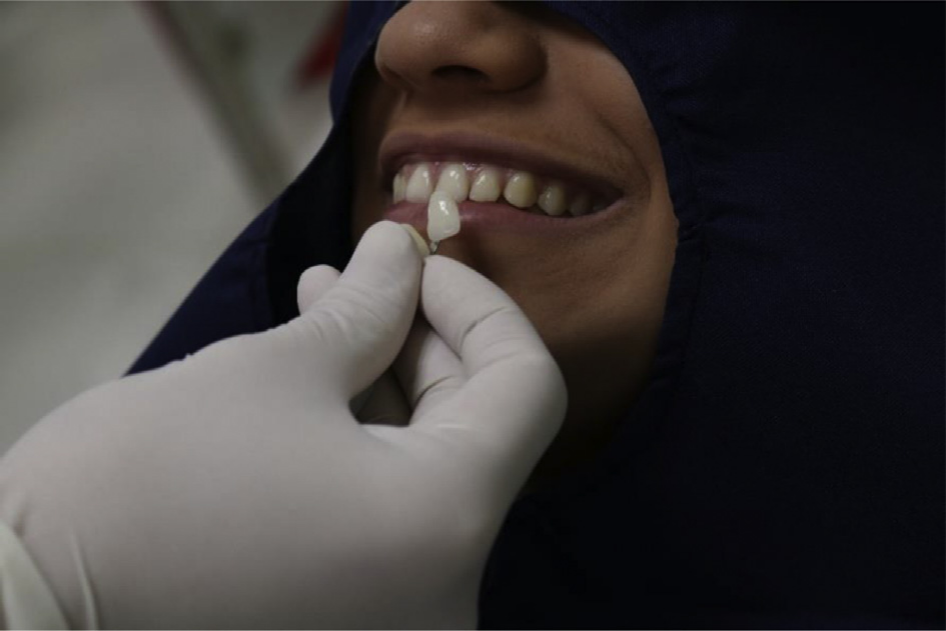
Visual method selection.

The study comprised three observation days for the visual method and three for the instrumental method. Patients were seated in dental chairs at the clinic, reclined at an angle of 45° to the floor, and numbered from 1 to 10 (Fig. 1). The patient arrangement was changed for each observation day. The patient's face was covered with a surgical drape to protect his or her identity and to avoid visual distractions and subsequent recognition (Fig. 2). During the first two days, the observations were performed under the same clinical studio ambient lighting (fluorescent ceiling light). On the third day, the ambient lighting was changed, and the observations were performed with the lights off and window curtains opened, so that sunlight was the only source of lighting (Table 1). The intensity of the lighting settings used was measured using a Lux meter (Dr. Meter LX1330B), and the observation days were separated by two weeks. It should be noted that the intensity of the light was not an object of investigation, as only the change from the initial ambient lighting setting was of interest. The examiners were asked to select the color of the right upper central incisor, specifically in the middle third portion, using the VITA Toothguide 3D-MASTER.

**Table 1:** Settings used for ambient lighting.

Clinical studio ambient lighting (1808 lux)		Sunlight inside the clinic (6921 lux)
Day 1	Day 2	Day 3
Baseline Measurement	Comparative Measurement	Comparative Measurement (Different ambient lighting)
(Same ambient lighting)		

**Fig. 3:**
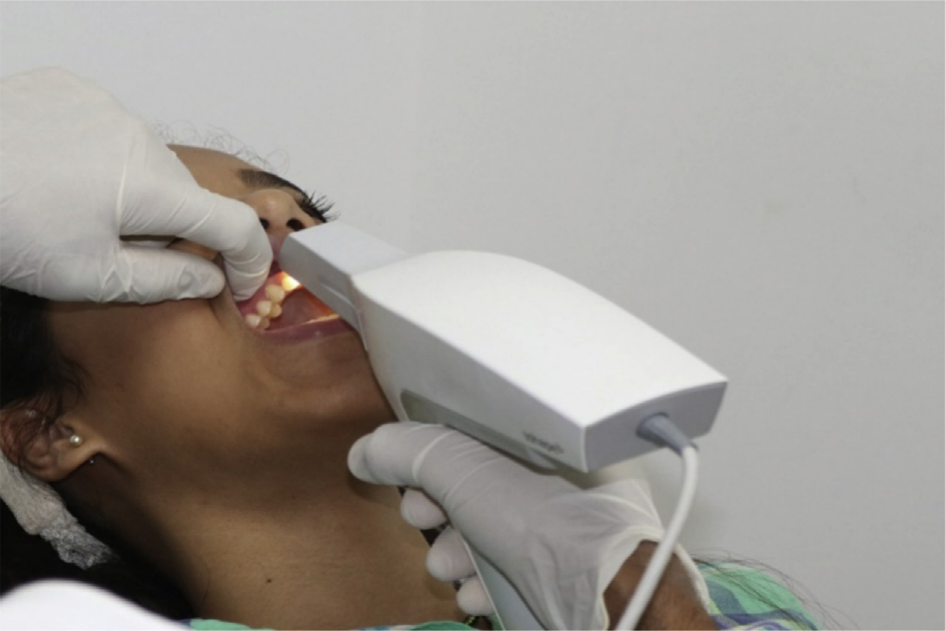
Intraoral scanner selection.

**Fig. 4:**
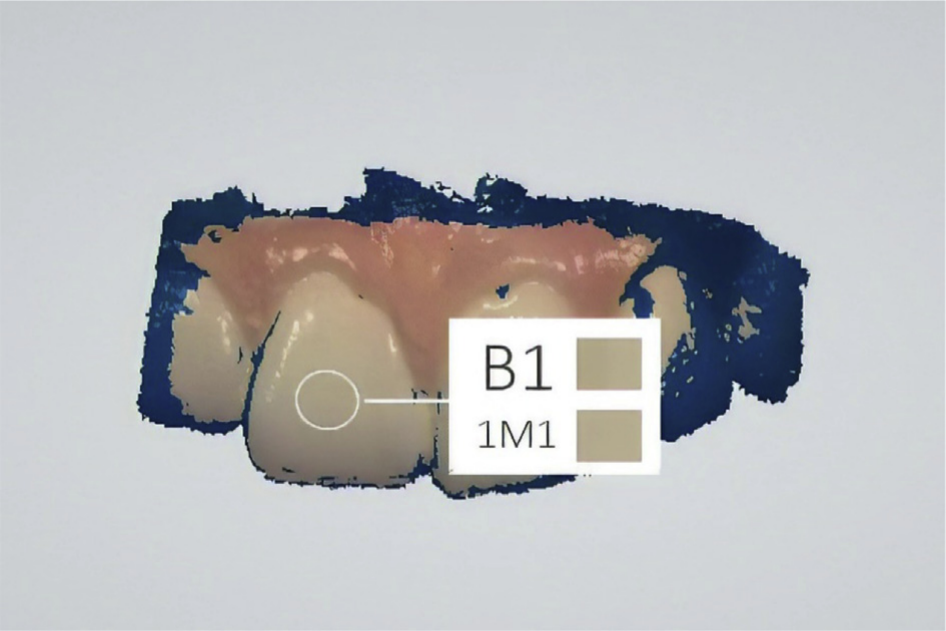
Shade selection for the middle third portion of the tooth using the TRIOS intraoral scanner, according to Vita 3D-Master Guide (lower code).

For the instrumental method, the same dental pieces as in the visual method were scanned using the 3Shape TRIOS intraoral scanner. The scanning was carried out using the same sequence of settings: two studio-light observations and one sunlight observation (Fig. 3). Observations were made every two weeks, with the scanner previously calibrated using the provided TRIOS Color Calibration Kit. After obtaining the scans, the colors were selected in the middle third portion of each tooth using the TRIOS software and the VITA Toothguide 3D-MASTER color codes as the reference values (Fig. 4).

A database was created using Microsoft Excel 2016. Matching measurements from days 1 and 2, with the same lighting, were paired and divided by the total number of measurements. Measurements taken on days 1 and 3, with different lighting, were processed in the same way. The average repeatability and standard deviation of each observer and the intraoral scanner was obtained, and each dimension was categorized individually.

For the statistical analysis, SPSS Statistics 23 software was used. To analyze the repeatability of each method a Student t-test was carried out. The same statistical test was used to compare the repeatability obtained by male and female examiners and to assess the repeatability according to the lighting used. To evaluate which group of examiners achieved a higher repeatability, a bivariate analysis of average contrast and standard deviation was implemented, and analysis of variance (ANOVA) was used to assess the statistical significance of the difference. Similarly, this statistical test was used to assess the repeatability according to the color dimension. The significance threshold was set at α = 0.05, corresponding to a 95% confidence level.

## 3 Results

**Fig. 5:**
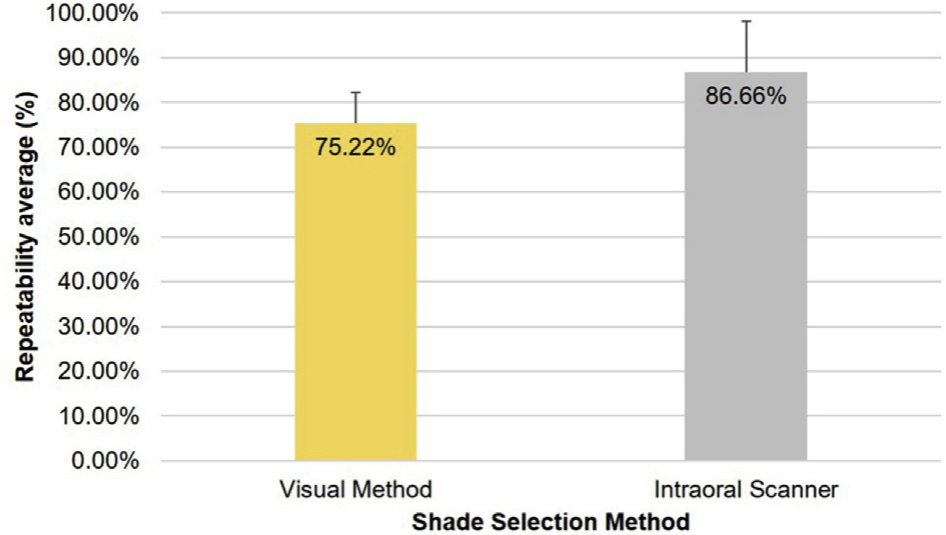
Comparison of repeatability in shade matching by the visual method and intraoral scanner.

As shown in Fig. 5, the intraoral scanner yielded the highest repeatability in shade matching, with 86.66% matching observations and a standard deviation (SD) of 11.48%. In contrast, the visual method yielded a repeatability of 75.22% (SD = 7.02%). The difference was statistically significant (*P* = 0.012).

**Fig. 6:**
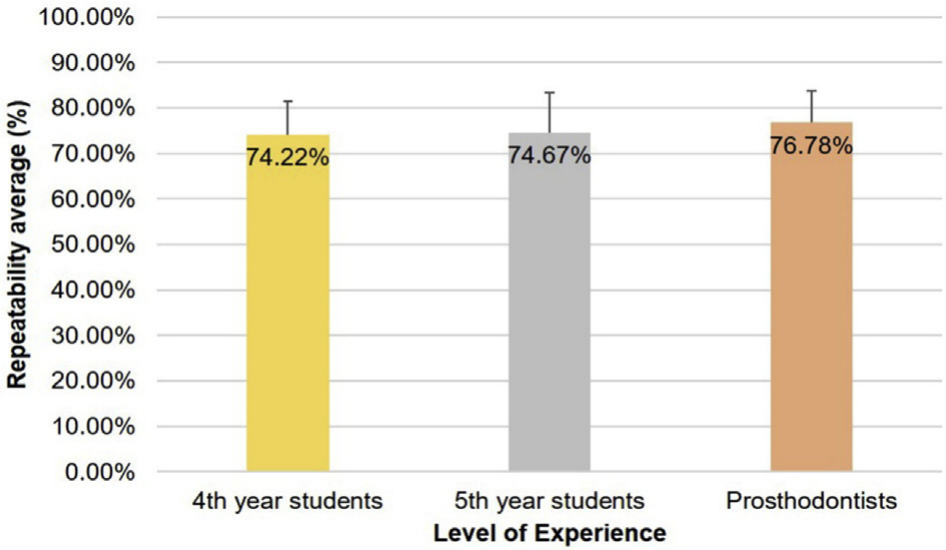
Repeatability in shade matching by the visual method of groups of observers classified by experience.

Fig. 6 shows the repeatability of visual shade matching according to expertise. The mean repeatability (SD) values for the three groups were as follows: 4th year students, 74.22% (7.20%); 5th year students, 74.67% (8.65%); and prosthodontists, 76.78% (7.02%); the group of prosthodontists showed slightly better performance, but the differences were not statistically significant according to ANOVA (F = 0.361, *P* = 0.700).

**Fig. 7:**
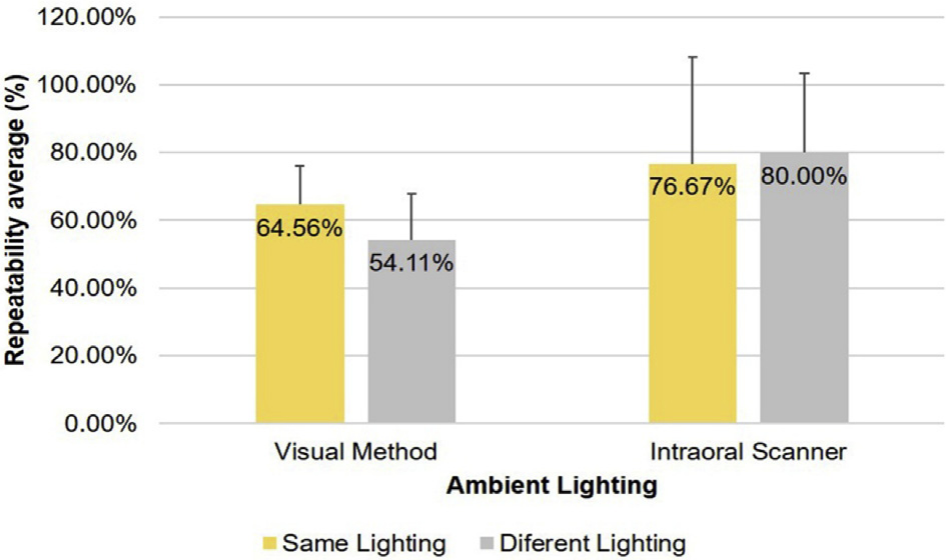
Repeatability in shade matching according to the ambient lighting used.

Regarding lighting conditions, as seen in Fig. 7, day 1 was taken as the baseline measurement with which the following days were compared. The visual method showed a difference of 10.45% between the two lighting settings used. Its repeatability decreased from 64.56% (SD = 11.63%), under the same ambient light setting as the baseline, to 54.11% (SD = 13.75%), when sunlight was used. The difference was statistically significant (*P* = 0.002). In contrast, the intraoral scanner exhibited increased repeatability from 76.67% (SD = 31.62%), with the same illumination, to 80.00% (SD = 23.31%), with different illumination, although this difference was not statistically significant (*P* = 0.792).

**Fig. 8:**
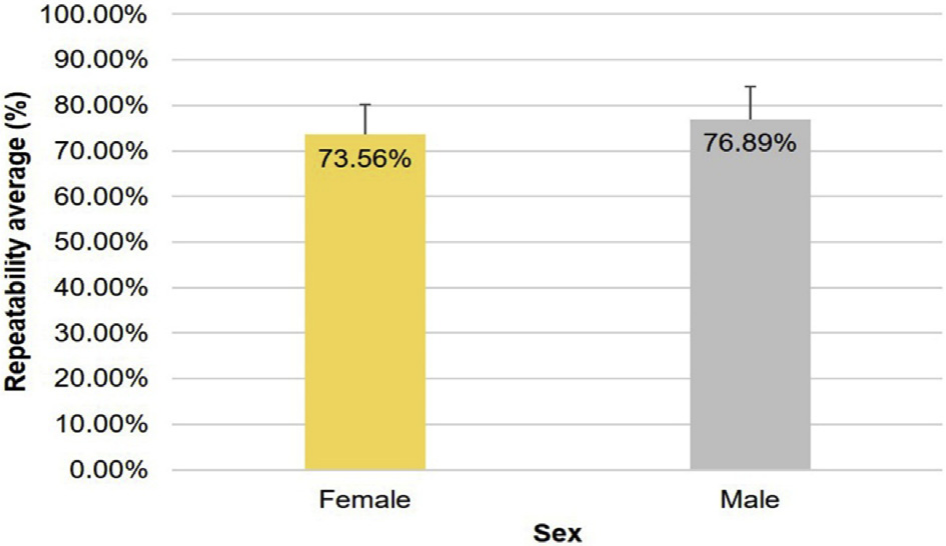
Repeatability in shade matching according to the sex of the observers.

Regarding the effect of sex on the repeatability in dental shade matching, Fig. 8 shows that there was no statistically significant difference between male and female examiners (*P* = 0.199). Male examiners achieved a repeatability of 76.89% (SD = 7.19%), while female examiners obtained a repeatability of 73.56% (SD = 6.69%).

**Fig. 9:**
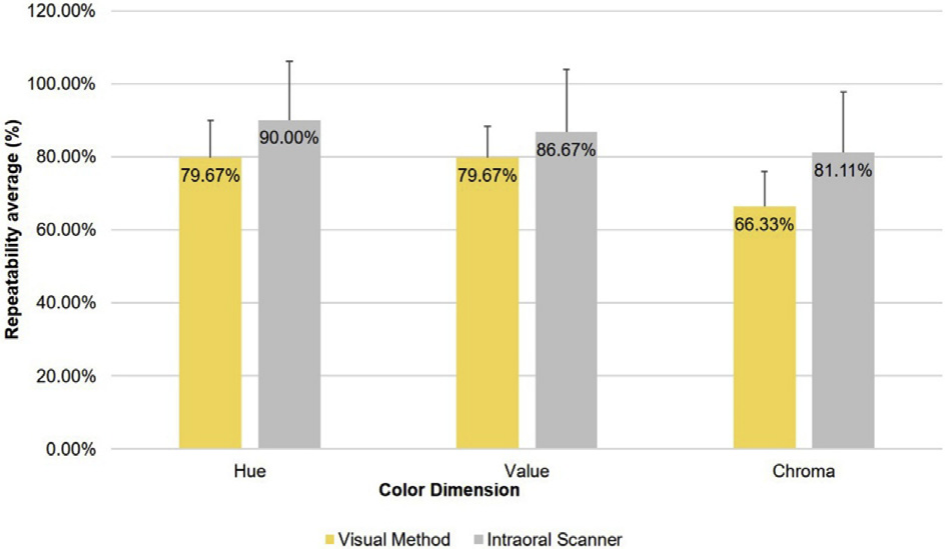
Repeatability of visual method and intraoral scanner in dental shade matching according to the three dimensions of color.

Repeatability, with regard to the three color dimensions, is shown in Fig. 9. For the visual method, value and hue dimensions reached the highest values (79.67% for both), with a standard deviation of 8.64% for value and 10.37% for hue. In contrast, when evaluating the chroma dimension, the visual method achieved a repeatability of 66.63% (SD = 9.68%), and the difference was statistically significant (F = 19.331, *P* = 0.000489).

The intraoral scanner achieved a repeatability of 90.00% (SD = 16.10%) in the hue dimension, but was less precise for the value and chroma dimensions, achieving a repeatability of 86.67% (SD = 17.21%) for value and 81.11% (SD = 16.60%) for chroma. Unlike the case of the visual method, these differences were not statistically significant (F = 0.728, *P* = 0.792).

## 4 Discussion

The results of this study confirm the superiority of the 3Shape Trios intraoral scanner over the visual method in terms of repeatability in dental shade matching. The three elements of the VITA Toothguide 3D-MASTER – hue, chroma, and value – were separately considered, and the ability of each observer to repeat the first choice of color was separately evaluated for the three color dimensions.

A means to determine with certainty the actual color of a dental piece is not yet available. The publications on dental colors take the results obtained by instrumental means, and in particular spectrophotometers, as a reference to evaluate the visual method [1, 33]. Other authors favor in vitro studies because the color of the porcelain sample or colored tab that is being evaluated is known with certainty [6, 8, 23, 25]. In contrast, the present study examined the repeatability of the visual method and the intraoral scanner by describing how frequently each method reproduced the same evaluation of the three color dimensions over time. To the best of our knowledge, this approach to measuring the repeatability of shade matching has yet to be reported in the literature, but it provides a tangible and quantitative means to measure and compare the repeatability of the methods.

Our findings confirm the superior repeatability of the 3Shape TRIOS intraoral scanner over visual matching, which has previously demonstrated in other studies [11, 34, 35, 36]. The examiners in our study were able to maintain their choices throughout the study in 75.22% of the cases, as compared with 86.66% of the intraoral scanner cases. The intraoral scanner results showed greater repeatability than those obtained by previous pioneering studies, such as that by Brandt and colleagues, who obtained a repeatability of 78.3% [35]. This difference found in our investigation was of statistical significance *(P* = 0.012).

Lighting was another factor that was evaluated to determine its influence in the shade matching repeatability of both the observers using the visual method and the results of the intraoral scanner. When using the same lighting source, the observers were able to maintain their choices regarding the base measurement in 64.56% of the cases—less than the 76.67% achieved by the intraoral scanner. In contrast, when changing the lighting with respect to the base measurement, the repeatability of the visual method was reduced to 54.11% *(P* = 0.002), while that of the intraoral scanner surprisingly increased to 80% (*P* = 0.792). In any case, it is evident that lighting has a direct effect on the ability of human observers to repeat a previously selected color choice.

Regarding the sex of the participants and its influence on the selection of dental color, previous studies have reported contradictory results, with some suggesting female superiority in performing the tasks [29] and others suggesting male superiority [30]. However, many investigations have cast doubt on the existence of such differences, and our results confirm the absence of a significant effect according to the observer's sex [22, 24, 25, 31, 32]. Indeed, while male observers achieved a mean repeatability of 76.89%, slightly higher than the 73.56% achieved by the female observers, but the difference was not statistically significant.

In terms of the color dimensions of hue, chroma, and value, Gómez-Polo et al. [33] found a correlation between visual and instrumental methods corresponding to a kappa coefficient of 0.6507 for value, followed by hue (0.4337) and finally chroma (0.3578), which is in agreement with the order of clinical importance of these three elements to obtain an adequate shade matching for dental prostheses. Using the visual method, equal repeatability (79.67%) was obtained for value and hue, and a repeatability of 66.33% for chroma. However, the data obtained for the value dimension are less variable than those obtained for the hue. These results show that the observers' repeatability is significantly different (*P =* 0.000489) according to the dimension studied. Despite this, the identified values allow the achievement of a good aesthetic result in the clinic, because the highest repeatability corresponds to the aesthetically most important dimension – the value [33].

The literature generally reports good correlations between visual and instrumental methods in terms of determining the value dimension [19, 33]. In our study, the intraoral scanner was more precise when choosing the hue (90%), followed by the value (86.67%) and finally the chroma (81.11%). Although this order differs from the order of clinical relevance of the three dimensions, it is compensated by a greater repeatability in all three dimensions, as compared with the visual method. However, no significant relationship was found between the repeatability of the scanner and the color dimension studied. In addition, it was observed that chroma was the color dimension characterized by the lowest repeatability in both methods; for the visual method, this finding is in agreement with the lower capacity of the human eye to distinguish this dimension of color [19].

Clinical experience, in theory, should affect the ability of the observers to choose a dental tone, because with practice and repetition observers would be expected to develop protocols allowing more precise and reproducible results over time. However, despite the vast literature supporting this hypothesis [6, 21, 22, 23], many other studies have refuted it [8, 24, 25, 26]. Our results also refute this hypothesis, because while repeatability increased with experience from fourth-year students to fifth year students to prosthodontists, the differences were minimal and not statistically significant.

The main strengths of this study include the following: (1) the in vivo setting that is similar to the clinical reality of the population and does not require inferences or extrapolations, and (2) the controlled environment in which the study was conducted, which minimized the effect of external factors. Another strength of the study is the consideration of each dimension of the color as an individual aspect. Selections were not considered as entirely correct or incorrect, but they were compared based on the three color dimensions. Finally, a novel aspect of this study is the use of an intraoral scanner in the selection of color because, to our knowledge, this device has not been widely tested in the literature [11, 35, 36]. In this respect, our study can be of benefit to the dental community.

In contrast, the limitations of the study include visual fatigue, which is a potential problem for all investigations related to dental shade matching, and it should be considered as a concern. The novel experimental design, while providing the advantages described above, makes comparisons with the previous literature more difficult. The lack of a reference instrument allowing the evaluation of accuracy together with repeatability is another limitation, since these are the two most important factors in an ideal measuring instrument. Other limitations include the small sample size and minor differences between professional groups in terms of experience. Moreover, factors such as the scan angle, time, distance and experience of the operator can influence the repeatability of the measurements. To overcome these limitations, future larger-scale in vivo studies using a similar experimental design and including a reference instrument to determine the accuracy of the measurements will be necessary to further expand the available knowledge concerning intraoral scanners and their application in dental shade matching.

## 5 Conclusions

The repeatability of the visual method was not affected by the experience or sex of the observer performing the selection. Lighting had a direct effect on the repeatability of the visual method for dental shade matching. The visual method reached the highest repeatability among color dimensions pertaining to the value, followed by hue and then chroma. The color dimensions measured by the intraoral scanner were not statistically different with regard to repeatability in dental shade matching.

The 3Shape Trios intraoral scanner showed higher repeatability than the visual method in shade matching for dental prostheses, as it was better able to maintain its observations over time, in a greater percentage, for all the three color dimensions. These findings suggest that this intraoral scanner can serve as a reference for dental health professionals and laboratory technicians. Despite the greater repeatability exhibited by this instrument, its accuracy was not investigated in this study, so that the instrument could in principle be affected by systematic errors leading to shade mismatches. Future studies will be needed to compare the performance of this device with other instruments used for dental shade matching in terms of both accuracy and repeatability.

## Declarations

### Author contribution statement

Juan Reyes, Pamela Acosta, Dalina Ventura: Conceived and designed the experiments; Performed the experiments; Analyzed and interpreted the data; Contributed reagents, materials, analysis tools or data; Wrote the paper.

### Funding statement

This research did not receive any specific grant from funding agencies in the public, commercial, or not-for-profit sectors.

### Competing interest statement

The authors declare no conflict of interest.

### Additional information

No additional information is available for this paper.
